# Effects of Long Chain Fatty Acid Synthesis and Associated Gene Expression in Microalga *Tetraselmis* sp

**DOI:** 10.3390/md12063381

**Published:** 2014-06-04

**Authors:** T. Catalina Adarme-Vega, Skye R. Thomas-Hall, David K. Y. Lim, Peer M. Schenk

**Affiliations:** Algae Biotechnology Laboratory, School of Agriculture and Food Sciences, The University of Queensland, Brisbane, Queensland 4072, Australia; E-Mails: t.adarmevega@uq.edu.au (T.C.A.-V.); s.thomashall@uq.edu.au (S.R.T.-H.); david.lim1@uqconnect.edu.au (D.K.Y.L.)

**Keywords:** nutrients, EPA, fatty acids, omega-3, gene expression

## Abstract

With the depletion of global fish stocks, caused by high demand and effective fishing techniques, alternative sources for long chain omega-3 fatty acids are required for human nutrition and aquaculture feeds. Recent research has focused on land-based cultivation of microalgae, the primary producers of omega-3 fatty acids in the marine food web. The effect of salinity on fatty acids and related gene expression was studied in the model marine microalga, *Tetraselmis* sp. M8. Correlations were found for specific fatty acid biosynthesis and gene expression according to salinity and the growth phase. Low salinity was found to increase the conversion of C18:4 stearidonic acid (SDA) to C20:4 eicosatetraenoic acid (ETA), correlating with increased transcript abundance of the Δ-6-elongase-encoding gene in salinities of 5 and 10 ppt compared to higher salinity levels. The expression of the gene encoding β-ketoacyl-coenzyme was also found to increase at lower salinities during the nutrient deprivation phase (Day 4), but decreased with further nutrient stress. Nutrient deprivation also triggered fatty acids synthesis at all salinities, and C20:5 eicosapentaenoic acid (EPA) increased relative to total fatty acids, with nutrient starvation achieving a maximum of 7% EPA at Day 6 at a salinity of 40 ppt.

## 1. Introduction

Long-chain polyunsaturated fatty acids (LC-PUFA), such as eicosapentaenoic acid (EPA), eicosatetraenoic acid (ETA) and docosahexaenoic acid (DHA), which are omega-3 fatty acids, and arachidonic acid (ARA), an omega-6 fatty acid, provide significant health benefits, including a reduced risk of hypertension, cardiac arrhythmia, myocardial infarction and thrombosis [1]. LC-PUFAs have also been found to have positive effects on brain function [2] and the healthy development of the foetal brain [3]. LC-PUFAs have primarily been extracted from small fatty marine fish, a limited resource, which hit peak production in the mid-1990s [4]. Concerns about the sustainability of LC-PUFA sources have increased, shifting research towards different sources, such as other marine organisms, transgenic plants and fungi. Interest on the sustainability of the omega-3 sources has moved efforts towards land-based production, including farmed fish, genetically modified plants, regulated krill catches and large-scale production of microalgae [4]. 

Microalgae are considered a viable and sustainable source of LC-PUFA, including omega-3 fatty acids. They have important advantages for commercial production over transgenic plants or fungi [5], including high areal productivity. They can also be grown on non-potable water and on non-arable land [6,7]. Microalgae have a natural adaptation capacity in diverse and even adverse environmental conditions. Some survival mechanisms include changing their chemical composition, such as modifying cellular fatty acid content to protect themselves from osmotic stress during rapid salinity changes, which may occur in natural environments, such as coastal rock pools [8,9,10,11,12,13,14]. The response to environmental stress of an altered salinity can lead to the cessation or slowing of algal growth and biomass accumulation, shifting photosynthetic energy towards the accumulation of chemical energy in the form of fatty acids (FA) or starch [10,15,16,17]. Marine species, like *Nannochloropsis* sp. [18] and *Dunaliella* sp. [19], can achieve a total lipid content of up to 47% and 60% of dry weight (DW), respectively, by modifying the light intensity, temperature and salinity during cultivation. Similarly, *Phaeodactylum tricornutum* was induced to enhance lipid content from 83.8 mg/g to 108.0 mg/g DW once the salinity of the media had been altered [17]. The response of microalgae to salinity stress is species-specific [20] and probably even strain-specific. Therefore, it is essential to investigate the effect of salinity on algal growth and omega-3 production in microalgal strains with commercial potential.

**Figure 1:**
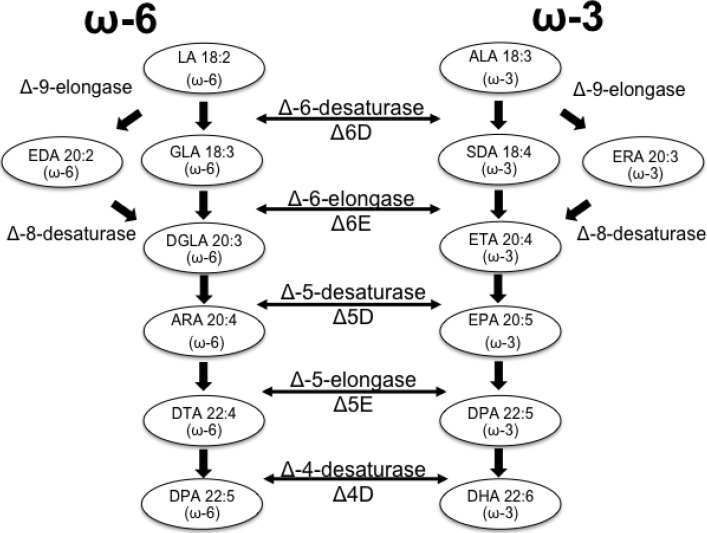
Biosynthesis of long-chain (LC)-PUFA’s via the conventional pathway.

Research on microalgal metabolic pathways has led to a better understanding of the mechanism for FA synthesis. Genes encoding enzymes involved in particular steps of FA synthesis have been sequenced and studied in diverse microalgal species. The traditional pathway for the synthesis of LC-PUFAs is presented in Figure 1. Most enzymes involved in the final steps of LC-PUFA biosynthesis and derivatization can either use omega-3 or omega-6 FAs as substrates. This pathway has been identified in animals, plants and microorganisms [21].

The synthesis of LC-PUFAs is largely regulated by a series of enzymes that can be classified in two groups: desaturases and elongases. The desaturases are a special group of oxygenases capable of removing hydrogen from a carbon chain, thus catalysing the formation of double bonds. Those enzymes use activated molecular oxygen to remove hydrogens from the carbon chain, creating a carbon/carbon double bond in the FA chain and a molecule of water [22]. The second enzymatic group involved in the synthesis of LC-PUFAs is responsible for increasing the length of the carbon chain and includes elongases [21]. To date, three types of elongases participating in the synthesis of PUFAs have been characterized: Δ6-elongase, Δ5-elongase and Δ9-elongase; each of these enzymes is substrate-specific. The elongation/desaturation reactions for LC-PUFA synthesis occur in two main pathways (Figure 1): the Δ6-desaturase/elongase and the Δ9-elongase/Δ8-desaturase; both use either linoleic acid (LA) for omega-6 FA or α-linoleic acid (ALA) for omega-3 FAs to make unsaturated fatty acid chains of 20 or more carbons [22].

In addition to the previously mentioned enzymes, there is another group of enzymes that can perform the elongation in the FA chain. They are known as microsomal FA elongation complexes. These enzymes mainly participate in the elongation of saturated or monounsaturated FA chains through four consecutive reactions of condensation, reduction, dehydration and a second reduction [23]. The first enzyme of the complex is the β-ketoacyl-coenzyme (BKAS), which catalyses the condensation of the acyl-CoA chain with malonyl-CoA. The additional three enzymes of the complex are 3-ketoacyl-CoA reductase, 3-hydroxyacyl-CoA dehydratase and enoyl-CoA reductase, which have been studied and characterized in yeast and *Arabidopsis thaliana* [23].

*Tetraselmis* species are green marine microalgae (Chlorophyta) commonly used in aquaculture, because of their high nutritional value. A number of species have been used as model organisms for physiological and biochemical studies, as well as for survival and adaptation mechanisms to diverse conditions, such as different salinities. Studies on salt tolerance and osmotic regulation have demonstrated that salinity provokes physiological changes, inducing several Na^+^-ATPase plasma membrane proteins in *Tetraselmis viridis* at high salinity [24]. Research on membrane pumps regulating the ionic flux in *Tetraselmis viridis* has shown that they are strongly involved in cytosolic homeostasis [25]. Studies on the expression of BKAS have found an increase of gene expression in *Dunaliella salina* as a result of salinity shifts from 0.5 to 3.5 M: this corresponded with an increased proportion of longer chain FAs in cell membranes [26]. Bioinformatics analyses decoding the microalgal genome have accelerated the identification of genes participating in the synthesis of molecules involved in microalgal survival mechanisms, such as osmoregulation proteins, as well as FA synthesis [26,27,28,29]. The identification of long-chain desaturases has given researches the ability to characterize and study their function in other organisms, such as yeast and plants [30,31,32]. Understanding FA synthesis in *Tetraselmis* sp. represents an important step towards the production of better nutritional quality microalgal strains for aquaculture in protein, as well as in the FA content and profile, including EPA and ARA. Little is known about the gene expression involved in the FA synthesis of *Tetraselmis* sp. as the salinity of the culture media changes. Therefore, the aim of this study was to profile FAs at various salinity levels in the marine microalga, *Tetraselmis* sp., and evaluate the expression of genes involved in the FA pathway and the osmotic balance for the synthesis of ETA and EPA.

## 2. Results

**Figure 2:**
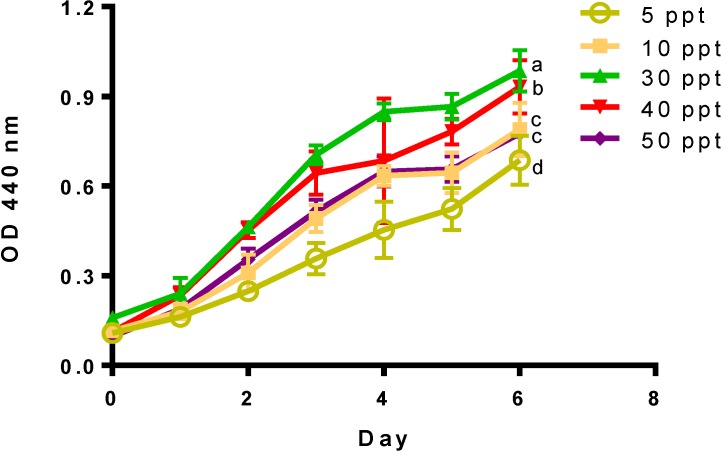
Optical density (440 nm) for *Tetraselmis* sp. cultivated at different salinities. Data represent mean values ± SDs for three independently grown cultures. Alphabets represent significant differences amongst salinities (*p* < 0.05).

This study evaluated the effect of different salinity levels (5 to 50 ppt) on pre-adapted cultures of *Tetraselmis* sp. M8. Cell density, nutrient consumptions, fatty acid profiling and expression differences for genes involved in FA synthesis were profiled over six days after the initial culture inoculation to determine the effect of salinity. This time period includes three growth phases: Days 0–2 (nutrient replete), Days 3–4 (nutrient deplete) and Days 5–6 (nutrient starved). As shown in Figure 2, salinity had a significant effect (*p* < 0.05) on the final day (Day 6) on cell density and growth rates (Table 1). A significant reduction in the final biomass (*p* < 0.05) was observed in high salinity cultures of 50 ppt, as well as in cultures grown at low salinities of 5 and 10 ppt. Cultures grown at 40 and 50 ppt, however, presented the highest growth rates measured during nutrient replete conditions (Table 2).

**Table 1:** List of genes and primers used for qRT-PCR.

Genes	Primers
Beta-Keto acyl synthase (BKAS)	5′-CAGGCCTTCGAGCATTTCTG-3′ 3′-GCGTCATATCAGGCGACAGC-5′
Delta-5-desaturase (Δ5D)	5′-TGGACGTTGGACATTGTAGGC-3′ 3′-CATTGTCATGCAGATTTGTGTACG-5′
Delta-6-elongase (Δ6E)	5′-CACCTACTACCTGCTTGCTGCC-3′ 3′-CTGGAACATTGTCAGGTAATGCC-5′
Acyl-CoA-synthase (ACSase)	5′-CACGTTGCTGTGCTTAATCTGC-3′ 3′-CGAGTGCAACCCTGAGGATATG-5′
Delta-5-elongase (Δ5E)	5′-TGAGGAAATGGTGCCAGCAG-3′ 3′-ACAAGTTCATCGAGTACCTCGACAC-5′
Glycerol-3-phosphate dehydrogenase (D3PDH)	5′-TCGTACCGCATCCACAAAGG-3′ 3′-GCTAAGGTGAAAGACAACGAGTCC-5′
Glucose-6-phosphate isomerase (G6Pi)	5′-GGGACAGCAGGTTATTGTGGAC-3′ 3′-TGCGCACCTTATCGGAGAAG-5′
Sodium ATPase (PyKPA)	5′-AAGGAAGCTGCGGATATGATTCTC-3′ 3′-TCAAGTTGTCAAAAATCAGACGACC-5′
Phosphate transporter (PHO)	5′-GACTTGGCACCCTTGAAGATAATG-3′ 3′-CTTACGCTCGCTCTTGGTGG-5′
3-ketoacyl-ACP reductase (KAR)	5′-CGGAGGAGATGTTAATGATGCG-3′ 3′-ATCAACCTCACCGGCGTCTT-5′
Delta-8-desaturase (Δ8D)	5′-GTCCGTAAAGGCTCCACTTCG-3′ 3′-GTATTTGACAAGACCACGCAGTTG-5′
Enoyl-ACP reductase (ENR)	5′-CTCCTTGACCTCAGTTGGGACA-3′ 3′-CTCAAACGGGTCCTTAATGGAGT-5′
Phosphatidic acid phosphatase (PP)	5′-TGTGGTCGGAGATCACATACGATA-3′ 3′-CAGTAGAGCGAGAACGACACCAG-5′
Delta-9-desaturase (Δ9D)	5′-GATATGAAAGCGTATGCCGAG-3′ 3′-GTAGCTCTAGCCGCCCCCTT-5′
Diacyl glycerol acyl transferase (DGAT)	5′-ATCAGAGGAACCTGTCCCATCA-3′ 3′-CTGCCATTTTTCACGAGCTAATG-5′
Beta-actin	5′-GCCTCAGAATCCCAAGACCAA-3′ 3′-GGCCTGGATCTGAACGTACATG-5′

**Table 2:** Specific growth rate (μ) and doubling time (d*t*) of *Tetraselmis* sp. cultures at different salinities.

Salinity (ppt)	Growth Rate (μ)	Doubling Time (d*t*)
5	0.414 ± 0.049	1.689 ± 0.207
10	0.487 ± 0.087	1.457 ± 0.287
30	0.532 ± 0.016	1.303 ± 0.038
40	0.695 ± 0.064 *	1.003 ± 0.088
50	0.644 ± 0.063 *	1.083 ± 0.105

Data represent mean values ± SDs for three independently grown cultures. * Indicates statistically significant differences (*p* < 0.05).

**Figure 3:**
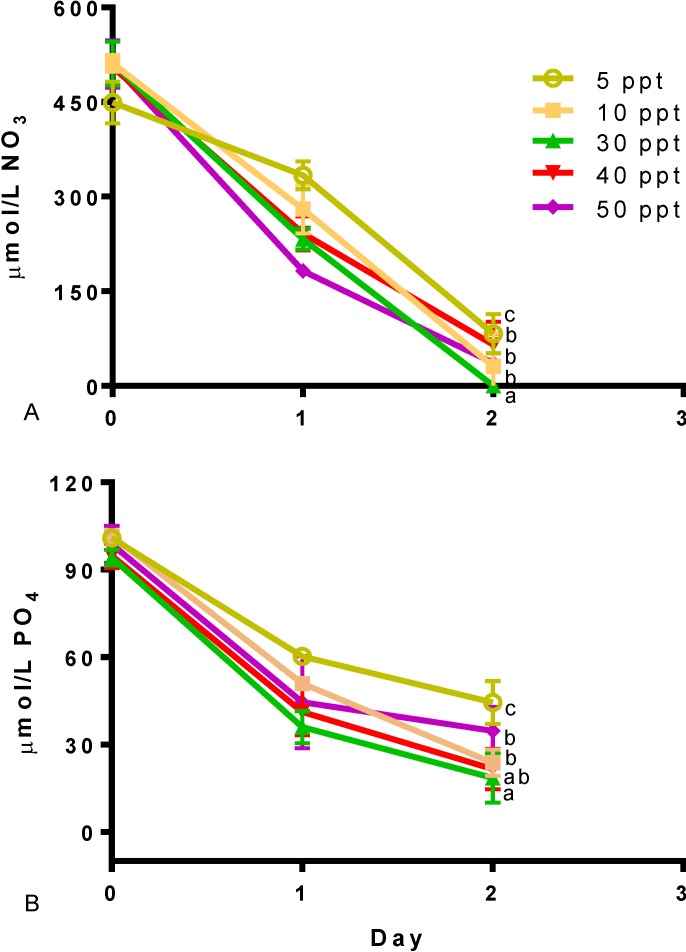
Nutrient draw down for different salinities in *Tetraselmis* sp. (**A**) Nitrate. (**B**) Phosphate. Data represent mean values ± SDs for three independently grown cultures. Letters represent statistically significant differences amongst salinities (*p* < 0.05).

Different salinity levels also had an effect on the nitrogen and phosphate consumption. Figure 3 presents the nutrient drawn down in *Tetraselmis* sp. M8 cultures. Statistically significant differences were found in the uptake of nitrogen and phosphorus (*p* < 0.05). Cultures at 5 ppt were the slowest in nutrient consumption. The fastest use of nutrients was found in culture grown at a salinity of 30 ppt (*p* < 0.05). Although cultures presented differences in nutrient consumption, all reached considerable N depletion within two days.

**Figure 4:**
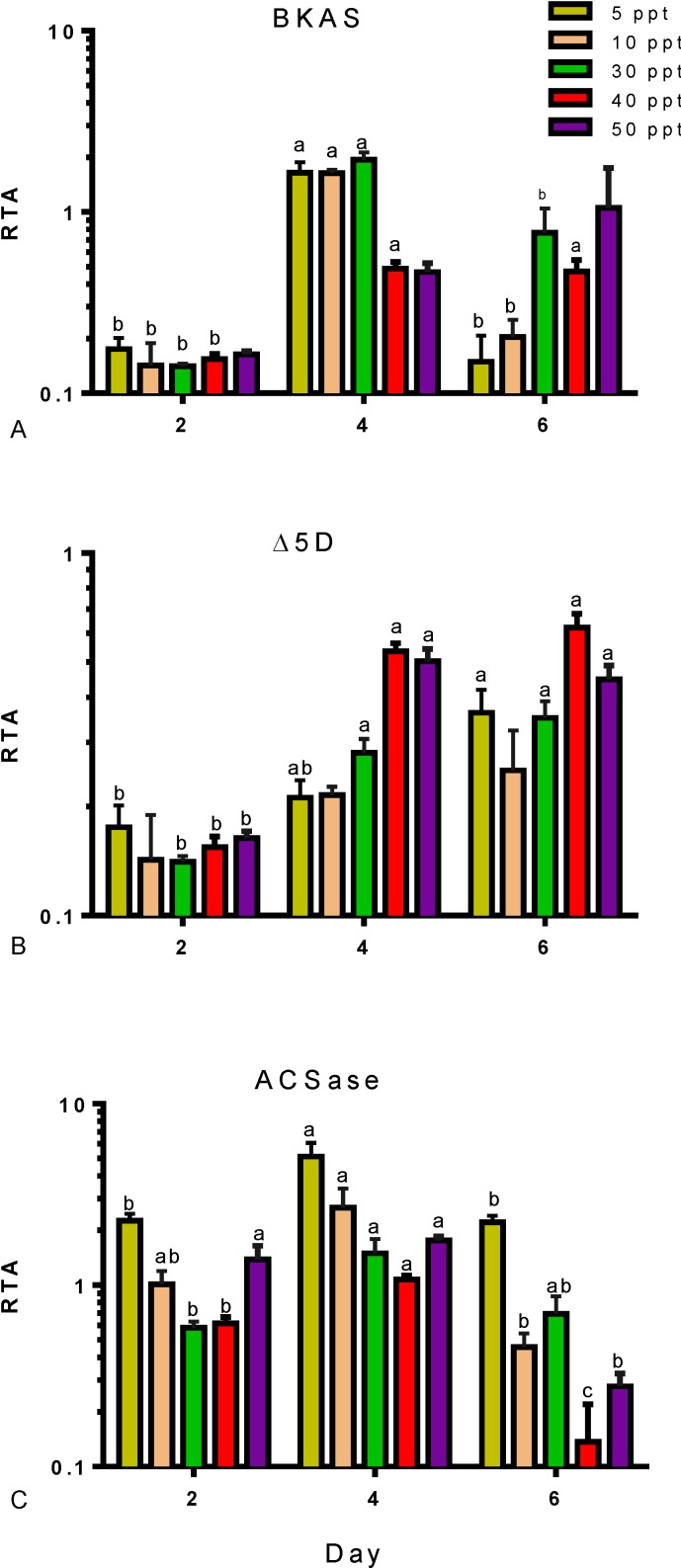
Expression profiles for three LC-PUFA biosynthesis genes in *Tetraselmis* sp., (**A**) BKAS, (**B**) Δ5D and (**C**) ACSase, under the influence of different salinities (5–50 ppt) and nutrient stress (Day 2, nutrient replete; Day 4, nutrient deplete; Day 6, nutrient starved). Transcript abundances are shown relative to *BETA-ACTIN* (RTA) measured by qRT-PCR from three independently grown cultures. Data represent mean values ± SDs. Letters represent statistically significant differences (*p* < 0.05).

**Figure 5:**
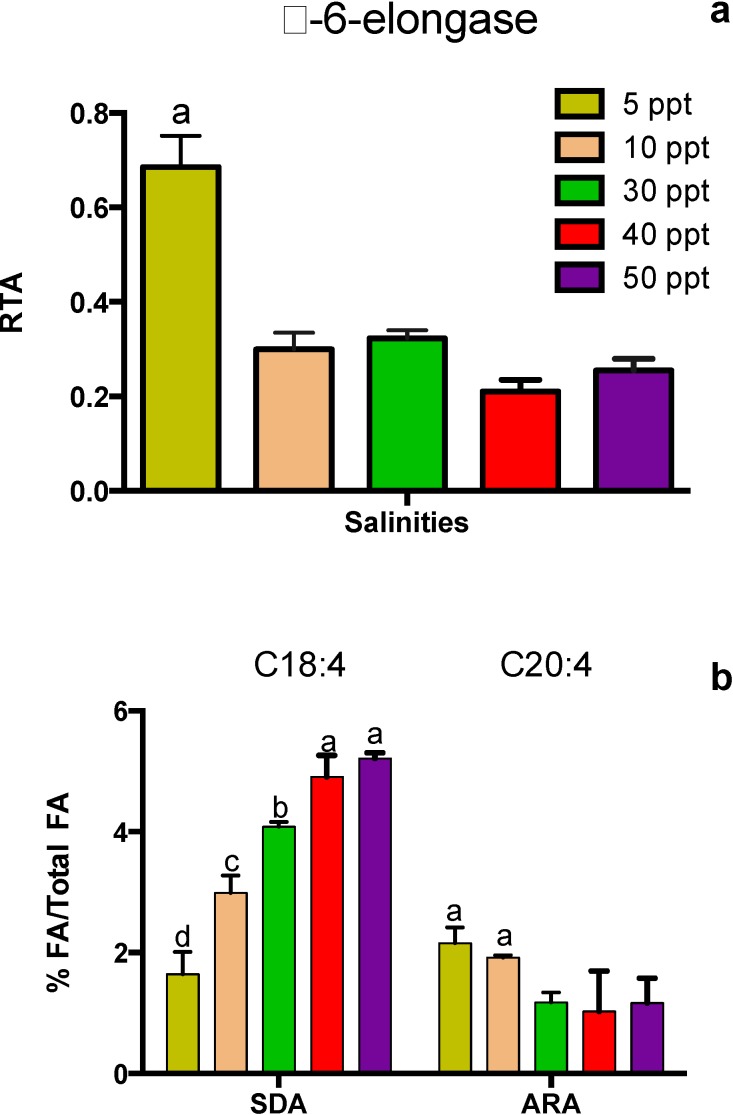
*Tetraselmis* sp. cultivated at different salinities on Day 4 after inoculation. (**A**) Expression profile for Δ-6-elongase-encoding gene. (**B**) Fatty acids C18:4 and C20:4 as a percentage of total FA. Data represent mean values ± SDs from three independently grown cultures. Letters represent statistically significant differences amongst salinities (*p* < 0.05).

Furthermore, the expression of fifteen genes involved in FA synthesis was analysed in *Tetraselmis* sp. cultivated at salinities of 5 to 50 ppt and under different nutritional conditions. Four genes, encoding BKAS, Δ5D, Δ6E and ACSace, were differentially expressed according to salinity and nutrient stress; these are presented in Figure 4 and Figure 5. Data of the remaining eleven genes are presented in Supplementary Table S1. The gene, *BKAS*, encodes an enzyme involved in the elongation of long-chain FAs by adding two carbons to the FA chain; its expression was significantly (*p* < 0.05) induced by nutrient deprivation (Figure 4A). On Day 4, the transcript levels were highest in low to medium salinities of 5, 10 and 30 ppt, and on Day 6, the expression was highest in medium to high salinities of 30, 40 and 50 ppt. The enzyme, Δ5D (Figure 1), catalyses the desaturation of C20:3 to C20:4 and of C20:4 to C20:5 in the omega-6 and omega-3 pathways, respectively. Expression of the gene encoding Δ5D (Figure 4B) increased with the progression of nutrient stress in all salinities. The upregulation of this gene correlates with EPA levels (Table 3). A consistent increase in percent of EPA was measured in cultures with nutrient depletion through to nutrient starvation. The expression of the ACSase-encoding gene in cultures with different salinities is presented in Figure 4C. Expression increased at all salinities with nutrient depletion (Day 4) and then decreased with nutrient starvation (Day 6).

The expression of the Δ6E-encoding gene and its relation to FA synthesis on Day 4 is presented in Figure 5. A salinity of 5 ppt led to the highest expression of this gene (*p* < 0.05), which also corresponds to increased efficiency for converting C18:4 to C20:4 (Figure 5B). There was a strong trend of increasing C18:4 and a moderate trend of decreasing C20:4 with increasing salinity. This corresponds to the trend of decreasing expression of the Δ6E-encoding *Tetraselmis* gene with increasing salinity. 

Fatty acid profiles for *Tetraselmis* sp. are shown in Table 3. The most abundant FAs were C16:0, C16:4, C18:3 (ALA), accounting for more than 50% of the total FA. The percentage of C16:0 increased in all salinities with a corresponding decrease in C18:3 (ALA) as nutrient stress progressed. There was no significant difference in the percentage of C20:5 (EPA) amongst the different salinities. There was, however, a significant increase (*p* < 0.05) in EPA content with nutrient stress, most notably at a salinity of 40 ppt. C20:4 (ETA) showed statistically higher accumulation at low salinities (5 and 10 ppt) and with nutrient starvation (Day 6) for all salinities.

**Table 3:** Fatty acid profile (%TFA) of *Tetraselmis* sp. cultivated at different salinities (5 ppt, 10 ppt, 30 ppt, 40 ppt and 50 ppt), under nutrient stress (Day 2, nutrient replete; Day 4, nutrient deplete; Day 6, nutrient starved).

Salinity															
Fatty Acids	5 ppt			10 ppt			30 ppt			40 ppt			50 ppt		
	2	4	6	2	4	6	2	4	6	2	4	6	2	4	6
C12:0	0.19 ± 0.07	0.23 ± 0.39	0.26 ±0.40	0.11 ± 0.05	0.11 ± 0.09	0.08 ± 0.09	0.15 ± 0.10	-	0.01 ± 0.01	0.06 ± 0.02	-	0.04 ± 0.07	0.09 ± 0.06	0.22 ± 0.21	0.05 ± 0.06
C14:0	0.38 ± 0.02	0.15 ± 0.27	0.32 ± 0.30	0.30 ± 0.01	0.23 ± 0.01	0.19 ± 0.09	0.23 ± 0.17	0.14 ± 0.04	0.17 ± 0.05	0.19 ± 0.06	0.11 ± 0.1	0.08 ± 0.07	0.24 ± 0.07	0.25 ± 0.22	0.17 ± 0.15
C14:1	1.22 ± 0.01	0.76 ± 0.10	0.72 ± 0.09	1.45 ± 0.05	1.11 ± 0.10	0.77± 0.16	1.67 ± 0.08	1.27 ±0.23	1.01 ± 0.16	1.66 ± 0.06	1.00 ± 0.48	0.65 ± 0.56	1.69 ± 0.05	1.27 ± 0.38	0.98 ± 0.28
C16:0	19.38 ± 0.01	21.22 ± 1.95	22.79 ± 1.36	18.14 ± 0.10	21.81 ± 0.58	22.73 ± 0.52	19.37 ± 0.90	22.15 ± 1.24	23.29 ± 1.00	18.38 ± 0.30	19.44 ± 1.88	20.99 ± 1.24	17.76 ± 1.21	18.70 ± 0.20	20.51 ± 1.83
C16:1	1.56 ± 0.11	1.31 ± 0.13	2.20 ± 1.29	3.63 ± 0.27	1.90 ± 0.07	1.627 ± 0.22	4.71 ± 0.76	2.36 ± 0.30	1.96 ± 0.32	4.26 ± 0.80	2.06 ± 0.80	1.72 ± 0.58	4.64 ± 0.55	2.72 ± 0.60	2.43 ±0.35
C16:3	6.51 ± 0.55	6.80 ± 0.77	5.89 ± 0.76	5.69 ± 0.18	5.58 ± 0.12	4.73 ± 0.33	4.33 ± 0.37	4.25 ± 0.09	3.44 ± 0.18	4.24 ± 0.25	4.70 ± 0.16	3.70 ± 0.12	3.94 ± 0.16	5.38 ± 0.14	4.68 ± 0.64
C16:4	16.54 ± 0.53	17.37 ± 4.48	13.80 ± 3.49	17.82 ± 0.01	15.02 ± 0.50	16.42 ± 1.61	19.01 ± 1.51	17.51 ± 1.36	17.16 ± 1.57	19.37 ± 0.17	22.26 ± 9.65	20.66 ± 4.87	19.47 ± 0.98	18.81 ± 4.11	18.37 ± 5.18
C18:0	-	-	-	-	-	-	-	-	-	-	-	-	-	-	-
C18:1	10.29 ± 0.38	11.94 ± 3.63	16.25 ± 2.80	7.54 ± 0.12	11.87 ± 0.78	13.30 ± 0.65	7.15 ± 0.79	10.49 ± 0.38	12.11 ± 0.32	6.36± 0.18	7.92 ± 3.44	7.12 ± 6.18	6.22 ± 0.36	8.65 ± 1.71	10.50 ± 1.72
C18:2	13.86 ± 1.40	15.15 ±0.88	14.39 ± 0.62	13.07 ± 0.27	15.55 ± 0.18	14.34 ± 0.19	11.37 ± 0.43	13.43 ± 0.46	12.81 ± 0.57	10.90 ± 0.45	13.20 ± 1.37	13.39 ± 0.34	9.81 ± 0.80	13.15 ± 0.16	13.01 ± 1.10
C18:3 (GLA)	0.61 ± 0.03	0.39 ± 0.10	0.79 ± 0.09	0.51 ± 0.01	0.631±0.040	0.81 ± 0.08	0.30 ± 0.18	0.47 ± 0.05	0.74 ± 0.06	0.32 ± 0.05	0.39 ± 0.34	0.74 ± 0.17	0.35 ± 0.05	0.49 ± 0.33	0.75 ± 0.14
C18:3 (ALA)	17.39 ± 1.31	14.35 ± 1.37	11.25 ± 0.79	18.76 ± 0.12	14.219±0.572	12.44 ± 0.37	18.77 ± 0.10	15.15 ± 0.52	13.99 ± 0.40	20.53 ± 0.30	16.19 ± 0.71	16.00 ± 2.21	20.51 ± 0.16	16.34 ± 0.60	14.69 ± 0.44
**C18:4**	2.26 ± 0.31	1.64 ± 0.37	1.41 ± 0.21	4.04 ± 0.18	2.99 ± 0.29	2.58 ± 0.21	4.84 ± 0.17	4.08 ± 0.08	3.62 ± 0.05	5.41 ± 0.33	4.90 ± 0.35	4.61 ± 0.84	5.95 ± 0.80	5.21 ± 0.09	4.33 ± 0.54
C20:0	-	-	-	0.46 ± 0.65	-	-	-	-	-	-	-	-	-	-	-
C20:1	1.67 ± 0.18	1.12 ± 0.21	1.38 ± 0.27	1.30 ± 0.03	1.21 ± 0.03	1.12 ±0.12	2.02 ± 0.26	1.64 ± 0.12	1.50 ± 0.05	2.08 ± 0.26	1.36 ± 0.60	1.12 ± 0.52	2.55 ± 0.36	1.90 ± 0.24	1.59 ± 0.10
C20:2	0.19 ± 0.26	0.33 ± 0.07	0.27 ± 0.24	0.18 ± 0.25	0.24 ± 0.03	0.31 ± 0.05	0.15 ± 0.14	0.29 ± 0.03	0.21 ± 0.18	0.21 ± 0.06	0.15 ± 0.13	0.18 ± 0.09	0.15 ± 0.14	0.33 ± 0.10	0.29 ± 0.02
C20:3	0.14 ± 0.09	-	-	0.10 ± 0.02	-	-	0.06 ± 0.10	-	-	-	-	-	0.05 ± 0.04	0.03 ± 0.04	-
**C20:4**	2.12 ± 0.16	2.15 ± 0.26	2.75 ± 0.24	1.63 ± 0.02	1.92 ± 0.04	2.20 ± 0.06	0.94 ± 0.30	1.18 ± 0.16	1.65 ± 0.12	1.06 ± 0.04	1.03 ± 0.67	1.73 ± 0.10	1.08 ± 0.07	1.17 ± 0.41	1.46 ± 0.29
**C20:5**	5.04 ± 0.11	5.06 ± 0.36	5.49 ± 0.32	4.91 ± 0.04	5.61 ± 0.10	6.13 ± 0.09	4.02 ± 0.43	5.01 ± 0.69	6.10 ± 0.49	4.44 ± 0.15	5.12 ± 0.91	7.18 ± 1.46	4.72 ± 0.44	5.29 ± 0.38	5.96 ± 0.30
SFA	19.96 ± 0.06	21.60 ± 2.59	23.37 ± 2.03	19.01 ± 0.79	22.15 ± 0.48	23.00 ± 0.53	19.76 ± 0.70	22.29 ± 1.26	23.46 ± 1.01	16.64 ± 0.24	19.55 ± 1.98	21.11 ± 1.34	18.09 ± 1.12	19.17 ± 0.36	20.74 ± 2.03
MUFA	14.74 ± 0.31	15.12 ± 3.98	20.55 ± 3.62	13.93 ± 0.23	16.10 ± 0.66	16.82 ± 0.96	15.56 ± 0.44	15.77 ± 0.61	16.58 ± 0.71	14.35 ± 0.56	12.34 ± 5.32	10.60 ± 7.83	15.11 ± 0.57	14.54 ± 2.88	15.50 ± 2.38
PUFA	64.66 ± 0.32	63.25 ± 6.52	56.04 ± 5.09	66.71 ± 0.07	61.76 ± 1.10	59.97 ± 1.48	63.68 ± 0.24	61.38 ± 2.00	59.74 ± 1.53	66.49 ± 0.41	67.95 ± 7.44	68.18 ± 9.24	66.02 ± 1.35	66.21 ± 3.18	63.55 ± 4.46
ω-3	26.81 ± 1.35	23.21 ± 1.66	20.89 ± 0.79	29.34 ± 0.25	24.73 ± 0.87	23.35 ± 0.65	28.57 ± 0.79	25.43 ± 1.14	25.37 ± 0.73	31.44 ± 0.22	27.24 ± 0.53	29.51 ± 4.40	32.25 ± 1.13	28.02 ± 0.75	26.44 ± 0.08

Data represent mean values ± SDs for three independently grown cultures. (-) undetected fatty acid. Total amounts of FAs are shown in Supplementary Table S2.

**Figure 6:**
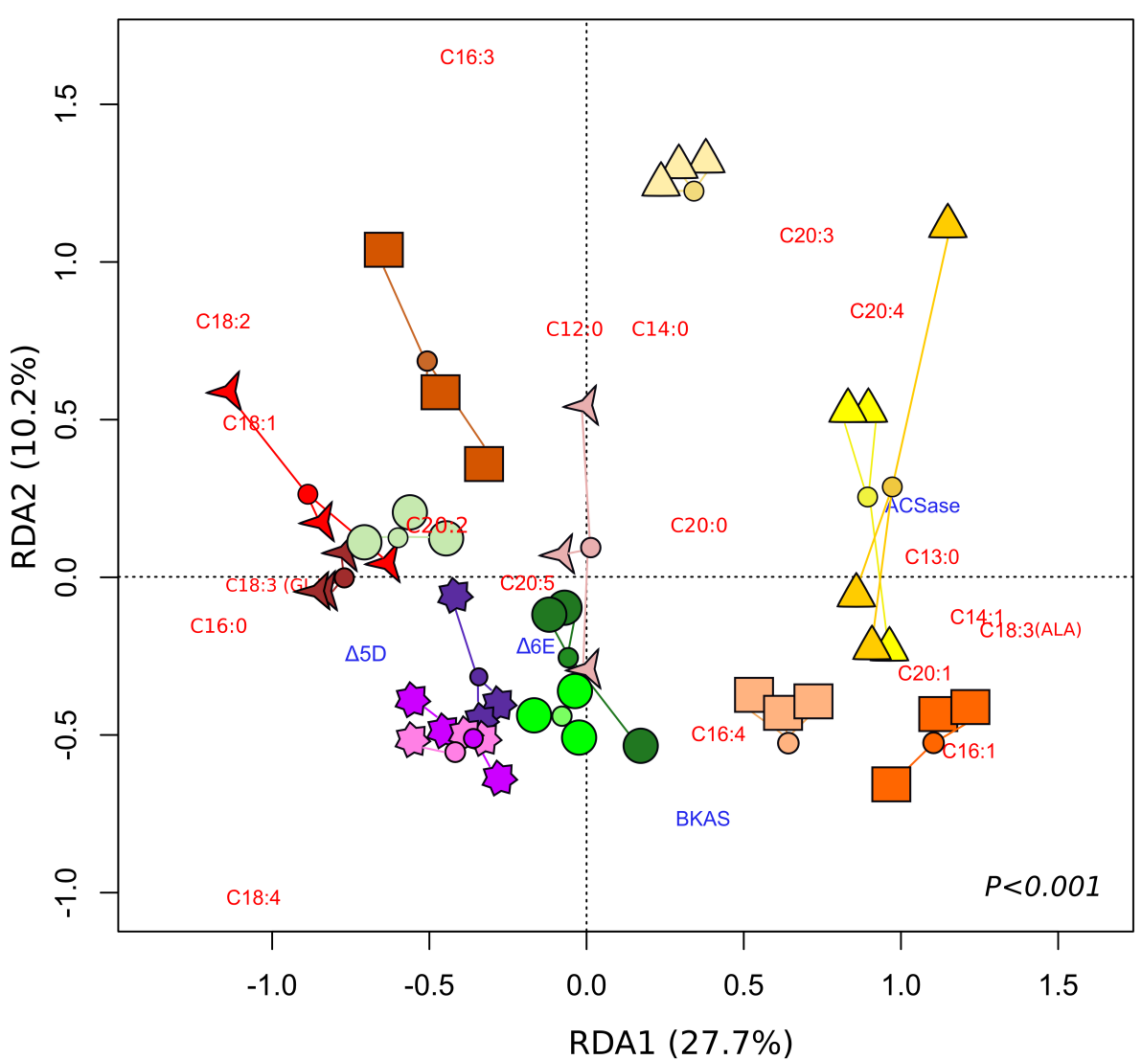
Redundancy analysis (RDA) summarizing the variation in gene expression and fatty acid production of *Tetraselmis* sp. at different salinities. (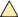) 5 ppt, Day 2; (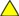) 5 ppt, Day 4; (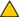) 5 ppt, Day 6; (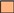) 10 ppt, Day 2; (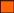) 10 ppt, Day 4; (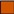) 10 ppt, Day 6; (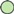) 30 ppt, Day 2; (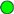) 30 ppt, Day 4; (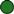) 30 ppt, Day 6; (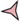) 40 ppt, Day 2; (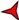) 40 ppt, Day 4; (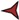) 40 ppt, Day 6; (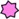) 50 ppt, Day 2; (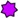) 50 ppt, Day 4, (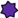) 50 ppt, Day 6. The small coloured circles represent the centroid of the treatment replicates.

Figure 6 presents the results of a redundancy analysis (RDA), which summarizes in two dimensions the variation of FA production and gene expression that can be attributed to the treatments applied. The primary (RDA1) and secondary (RDA2) axes of the RDA explain 27.7% and 10.2% of this variation, respectively. The gene expression of BKAS, Δ6E, Δ5D and ACSase-encoding genes, as well as FA proportions explained the difference amongst salinity treatments (RDA, Figure 6, *p* < 0.001). 

Different treatments showed different proportions of certain FAs and transcripts. A clear separation of treatments with a low salinities of 5 ppt (Days 2, 4, 6) and 10 ppt (Days 2, 4) and high salinities of 30 ppt, 40 ppt and 50 ppt was revealed along the primary axis. An induction of the ACSase-encoding gene was observed in salinities of 5 ppt relative to 40 and 50 ppt (Figure 6). The main differences between low and high salinity treatments are particularly attributed to the separation of Fas, such as C18:4 and C20:4 (Table 3), which were present at higher proportions in the 5 ppt and 40–50 ppt treatments, respectively. It can also be noted that these particular FAs are located in contrasting quadrants along both primary and secondary axes of the RDA (RDA1 and RDA2).

On the secondary axes (RDA2), the main differences were observed between salinities of 5 ppt at Day 2 and 10 ppt at Day 4, which was clearly shown by FAs C16:3 and C18:4 (Figure 6). Differences between low and high salinities were also observed. Salinities of 40 and 50 ppt led to higher proportions of C18:4 relative to 5 ppt, which presented lower percentages of C18:4, but higher C20:4 values.

## 3. Discussion

*Tetraselmis* sp. was used as a laboratory model strain to study the effect of salinity on growth, FA accumulation and the expression of genes involved in the FA synthesis. Optimal growth conditions are species-specific depending on cellular adaptation mechanisms to environmental stress. This study found that *Tetraselmis* sp. M8 displayed the best growth rate at 40 ppt, but the highest final biomass at 30 ppt. Other studies found that *Tetraselmis suecica* presented a maximum cell density of 6.4 × 10^6^ cell/mL at a salinity of 25 ppt [33]. Diverse microalgal species have been found to have optimal growth when cultured at different salinities. For example, *Chaetoceros wighamii* [10] and *Gracilaria corticata* [34] presented their highest growth rate at 25 and 35 ppt, respectively. *Nannochloropsis* sp. showed a high growth rate at 13 ppt when cultured at low light irradiance (170 μmol photon/m^2^ s); however, when *Nannochloropsis* sp. was cultivated under high light irradiance (700 μmol photon/m^2^ s), its best growth occurred at 27 ppt [18]. Halotolerant microalgal species *D. salina* demonstrated the highest cell concentration at 1.0 M NaCl (58 ppt) [19]. Although there are several studies related to salt tolerance in microalgal species, the salt tolerance mechanism in several species of commercial interest, such as *Tetraselmis* sp., requires further study. Depending on the strain and its salinity tolerance, FA synthesis can be induced or inhibited. *D. salina* was found to increase its FA content from 60% to 67% when salinity was elevated from 0.5 to 1 M NaCl (58 ppt) [19]. In the present study, *Tetraselmis* sp. M8 was observed to have the highest omega-3 FA proportion in relation to total FAs at Day 2 (nutrient replete) at a salinity of 50 ppt, followed by 40 and 30 ppt. Omega-3 Fas, such as EPA, have been associated with high growth due to their importance in cellular functions. Studies on *Pinguiococcus pyrenoidosus* demonstrated that maximum EPA and DHA production occurred at salinities of 30 ppt [35]. *Schizochytrium limacinum* was found to have high growth rates at salinities between 18 and 27 ppt, while its highest DHA content was found at a salinity of 9 ppt after five days of cultivation [36]. Although the highest EPA production is more commonly associated with nutrient replete conditions optimal for cellular growth, not all species of microalgae have higher ratios of this FA during logarithmic growth. The present study, for example, showed that the proportion of EPA in *Tetraselmis* sp. increased during the progression of nutrient stress. However, total omega-3 FAs decreased with nutrient stress, primarily due to the reduction of ALA.

Gene expression for FA synthesis has previously been studied in several microalgal species, demonstrating that up- and/or down-regulation of genes occurs as a result of the changes of external conditions, like salinity [37,38]. Growth is promoted when cultures are under nutrient replete conditions, enhancing gene expression by using large amounts of anabolic structural components. However, once nutrients are depleted, autophagic processes can be activated to provide intracellular nitrogen for limited *de novo* synthesis, allowing cells to change and adapt [37]. Gene expression for the LC-PUFA synthesis pathway was generally upregulated by nutrient deprivation (Figure 4). Differential gene expression for Δ5D, involved in the desaturation of FA chains for the synthesis of ARA and EPA, was higher at high salinity once nutrients were depleted from the media (Figure 4B). On the other hand, lower salinity levels induced higher expression of the Δ6E-encoding gene, involved in the elongation of C18:4 into C20:4 and C18:3 into C20:3 in the omega-3 and omega-6 FA pathways, respectively (Figure 1). Although enzymes have been reported to have a dual activity in each FA pathway; the little or undetectable C20:3 in *Tetraselmis* sp. FA profiles indicates that the omega-3 FA pathway is more likely to be used than the omega-6 pathway. 

A salinity shock experiment in *D. salina* found that the proportion of 18, 20 and 22 carbon FAs and desaturation were higher at high salinity (3.5 M; 203 ppt) compared to normal salinity (0.5 M; 29 ppt), which had a higher proportion of saturated 16 carbon FAs. The BKAS-encoding gene was also highly induced with the high salinity treatment. It was therefore suggested that the BKAS elongation reactions provided a sufficient substrate for long-chain desaturases to work. Therefore, salinity can activate FA modification by the elongation and desaturation of FA chains to contribute to the osmoregulation of the salt tolerance of microalgae [26]. In *Tetraselmis* sp., we found an increase in BKAS and ACSase transcript abundance with nutrient depletion, but there was no significant difference in gene expression between salinities and no differences in the proportion of FA carbon chain length or the level of desaturation. The differences we found in the current experiment were probably due to the pre-adaptation to salinity, rather than osmotic shock.

## 4. Methods

### 4.1. Algae Culture and Cultivation Conditions

*Tetraselmis* sp. (strain M8) was isolated from the south-east coast of Queensland, Australia (26°39′39″ S 153°6′18″ E), and stored in the culture collection of the Algae Biotechnology Laboratory at The University of Queensland [39]. Prior to the experiment, the algal strain was pre-adapted in f/2 silicate-free medium [40] that was phosphate enriched (100 μM), with the salinities to be tested using artificial sea water (Acuasonic Ocean-Nature sea salt). The culture in the logarithmic phase was used as inoculum; inoculation concentration varied slightly depending on the starter culture optical densities. Approximately 20 mL of each pre-adapted algal stock culture were transferred to 180 mL of enriched f/2 medium in a 250-mL Erlenmeyer conical flask with artificial salty water adjusted to 5, 10, 30, 40 and 50 parts per thousand (ppt) using three independently-grown cultures. Salinity was determined using a Reed TDS salinity conductivity meter (Toronto, ON, Canada). Cultures were incubated at 25 °C under a 16/8 h light/dark cycle (90 μmol/m^2^/s fluorescent lights) regime with constant bubbling. Optical density (OD) 440 nm measurements were performed daily to monitor the growth rate. Nitrogen and phosphorus contents were determined from Day 0 until nutrient depletion. Samples for FA profiling and RNA extraction were collected on Day 2 (nutrient replete), Day 4 (nutrient deplete) and Day 6 (nutrient starved).

### 4.2. Culture Media Nutrient Analysis

Total nitrate was measured using the commercial colorimetric API Aquarium Pharmaceutical Nitrate NO_3_^−^ test kit; colour intensity was measured using a spectrophotometer at a wavelength of 545 nm. A standard curve was generated and used to determine nitrate concentrations in medium samples (algae were previously removed by centrifugation); 0–300 μM was found to have a linear colorimetric relationship to the NO_3_^−^ concentration. Total phosphate was determined using the colorimetric API Aquarium Pharmaceutical Phosphate PO_4_^3−^ test kit; colour intensity was measured using a spectrophotometer at a wavelength of 690 nm. A standard curve was generated; 0–60 μM was found to have a linear colorimetric relationship to the PO_4_^3−^ concentration. 

### 4.3. Fatty Acid Analysis

Fatty acids were quantified by gas chromatography-mass spectrometry (GC/MS) by Metabolomics Australia at the University of Western Australia, as described previously [41], with the exception that 5 mg of culture was used instead of 2 mL of culture. Hydrolysis and methyl-esterification was performed, as described previously [41].

### 4.4. Total RNA Extraction and cDNA Synthesis

Total RNA from microalgal biomass was extracted using the SV Total RNA Isolation System (Promega, Madison, WI, USA) using centrifugal pellets obtained from 15 mL of culture. RNA concentrations were measured with a Qubit^®^ 2.0 Fluorometer (Invitrogen, Carlsbad, CA, USA). One microgram of total RNA was used for cDNA synthesis using the Superscript III reverse transcriptase (Invitrogen, Carlsbad, CA, USA) for quantitative reverse transcriptase PCR following the manufacturer’s instructions. 

### 4.5. Real-Time Quantitative Reverse Transcriptase PCR

Primers used for real-time quantitative reverse transcriptase PCR (qRT-PCR) were designed using Primer Express software (Applied Biosystems, Foster city, CA, USA), based on a recently generated draft transcriptome of *Tetraselmis* sp. by the Algae Biotechnology Laboratory at The University of Queensland, Brisbane, Australia (transcriptome data will be published elsewhere) [42]. Primers were designed in conserved regions to cover the majority of gene family members. Each reaction was performed in a final volume of 10 μL and contained 1 μL of the cDNA (1:4 diluted), 1 μL of each primer (1 μM) and 5 μL SYBR Green using the 7900 HT Fast Real-time PCR system (Applied Biosystems, Foster City, CA, USA). *Tetraselmis* sp. M8 transcript levels were normalized to the expression of *β-ACTIN*. Thermal cycling conditions consisted of 10 min at 95 °C and 45 cycles of 15 s at 95 °C and 1 min at 60 °C prior to 2 min at 25 °C.

### 4.6. Statistical Analyses

The analysis of variance (ANOVA) was performed using the Statistical Package for the Social Sciences (SPSS, IMB, New York, NY, USA). All significant differences (*p* < 0.05) amongst values obtained for different salinities and harvesting times were determined using the Tukey HSD test. Redundancy analysis (RDA) was performed using the package, vegan, implemented in R 3.0.2 [43].

## 5. Conclusion

Changes in salinity primarily altered biomass productivity, with 30 and 40 ppt having the highest growth rate and final productivity. Salinity had no significant effect on the percentage of EPA or total FA production (Supplementary Table S2). Under nutrient depletion, most of the genes analysed from the FA synthesis pathway were strongly upregulated, but the gene expression typically decreased once cultures were fully starved (Supplementary Table S1). A correlation between the upregulation of the Δ6E-encoding gene and the conversion of C18:4 to C20:4 was found, indicating that the omega-3 pathway is more likely to be used for the synthesis of LC-PUFAs in *Tetraselmis* sp. Additionally, an increase of EPA (C20:5) and ETA (C20:4) proportions with the progression of nutrient stress was found, especially algal cultures grown at 40 ppt of salinity. However, the trigger for the synthesis of EPA still remains unclear. Hence, further studies are required to determine the responsible factors for the upregulation of the omega-3 pathway synthesizing EPA in *Tetraselmis* sp. under diverse environmental conditions.

## Supplementary Files

Supplementary File 1 Supplementary Information (PDF, 78 KB)
