# Supplementary material for: Effects of Long Chain Fatty Acid Synthesis and Associated Gene Expression in Microalga Tetraselmis sp
Source: Mar Drugs. 2014 Jun 4;12(6):3381–98. doi: 10.3390/md12063381 (PMC4071582; doi:10.3390/md12063381)
Supplement: Supplementary File 1 — Supplementary Information (PDF, 78 KB) [file marinedrugs-12-03381-s001.pdf]

Supplementary Information

Table S1. Expression profiles for 15 genes in *Tetraselmis* sp. Transcript abundances are shown relative to *ACTIN* measured by qRT-PCR from three independent biological replicates. Data represent mean values ± SDs.

	**Salinity**								
**Genes**	**5**			**10**			**30**		
	**2**	**4**	**6**	**2**	**4**	**6**	**2**	**4**	**6**
BKAS	0.149 ±0.028	1.649 ±0.404	0.149 ±0.101	0.166 ±0.078	1.639 ±0.130	0.204 ±0.086	0.379 ±0.325	1.947 ±0.328	0.768 ±0.480
Δ5D	0.175 ±0.045	0.212 ±0.043	0.363 ±0.098	0.142 ±0.081	0.215 ±0.021	0.252 ±0.125	0.141 ±0.009	0.281 ±0.045	0.351 ±0.068
Δ6E	0.051 ±0.007	0.686 ±0.115	0.174 ±0.073	0.017 ±0.010	0.300 ±0.061	0.107 ±0.024	0.054 ±0.066	0.323 ±0.030	0.035 ±0.029
ACSase	0.270 ±0.355	5.113 ±1.665	2.226 ±0.316	1.014 ±0.311	2.676 ±1.257	0.457 ±0.148	0.583 ±0.082	1.502 ±0.515	0.695 ±0.303
Δ5E	0.029 ±0.011	0.644 ±0.767	0.074 ±0.086	0.015 ±0.009	0.119 ±0.018	0.029 ±0.002	0.105 ±0.156	0.135 ±0.033	0.217 ±0.062
D3PDH	0.040 ±0.007	0.184 ±0.102	0.065 ±0.016	0.035 ±0.014	0.079 ±0.026	0.064 ±0.045	0.278 ±0.403	0.096 ±0.024	0.160 ±0.163
G6Pi	0.368 ±0.039	0.364 ±0.084	0.392 ±0.184	0.252 ±0.110	0.296 ±0.040	0.141 ±0.057	0.533 ±0.398	0.599 ±0.262	0.648 ±0.373
PyKPA	1.920 ±0.796	9.048 ±0.878	7.455 ±3.141	1.691 ±0.803	14.376 ±2.968	3.208 ±2.273	6.920 ±7.919	27.129 ±8.719	16.692 ±7.390
PHO	0.128 ±0.031	0.212 ±0.093	0.310 ±0.162	0.071 ±0.059	0.253 ±0.099	0.388 ±0.146	0.516 ±0.818	0.458 ±0.127	0.812 ±0.627
KAR	0.117 ±0.070	0.272 ±0.057	0.077 ±0.057	0.062 ±0.037	0.057 ±0.018	0.063 ±0.068	0.109 ±0.118	0.077 ±0.046	0.198 ±0.219
Δ8D	0.336 ±0.097	0.728 ±0.072	0.861 ±0.327	0.304 ±0.102	1.373 ±0.228	0.835 ±0.402	0.350 ±0.058	2.266 ±0.582	1.672 ±1.165
ENR	0.494 ±0.287	0.647 ±0.080	0.130 ±0.058	0.207 ±0.061	0.431 ±0.103	0.089 ±0.022	0.157 ±0.135	0.328 ±0.055	0.649 ±0.293
PP	0.136 ±0.023	0.162 ±0.068	0.091 ±0.041	0.108 ±0.087	0.150 ±0.018	0.113 ±0.026	0.596 ±0.878	0.161 ±0.073	0.239 ±0.169
Δ9D	0.795 ±0.071	0.950 ±0.113	0.701 ±0.335	0.601 ±0.304	0.504 ±0.046	0.272 ±0.129	1.129 ±1.509	0.564 ±0.078	0.516 ±0.470
DGAT	0.093 ±0.009	0.468 ±0.041	0.240 ±0.077	0.093 ±0.015	0.247 ±0.075	0.131 ±0.040	1.142 ±1.865	0.134 ±0.017	0.171 ±0.060

Table S1. *Cont.*

	**Salinty**					
**Genes**	**40**			**50**		
	**2**	**4**	**6**	**2**	**4**	**6**
BKAS	0.223 ± 0.005	0.0487 ± 0.073	0.471 ± 0.126	0.774 ± 0.130	0.467 ± 0.096	1.052 ± 1.209
Δ5D	0.154 ± 0.019	0.536 ± 0.049	0.623 ± 0.096	0.164 ± 0.013	0.503 ± 0.069	0.048 ± 0.072
Δ6E	0.015 ± 0.004	0.210 ± 0.042	2.046 ± 0.491	0.100 ± 0.014	0.255 ± 0.043	16.182 ± 24.053
ACSase	0.616 ± 0.087	1.073 ± 0.095	0.137 ± 0.145	1.387 ± 0.458	1.765 ± 0.168	0.277 ± 0.086
Δ5E	0.039 ± 0.012	0.046 ± 0.018	0.113 ± 0.026	0.036 ± 0.011	0.020 ± 0.007	0.171 ± 0.076
D3PDH	0.066 ± 0.002	0.065 ± 0.007	0.054 ± 0.025	0.103 ± 0.016	0.070 ± 0.020	0.303 ± 0.378
G6Pi	0.428 ± 0.028	0.528 ± 0.062	0.638 ± 0.256	0.546 ± 0.053	0.644 ± 0.064	0.666 ± 0.514
PyKPA	1.924 ± 0.229	19.629 ± 3.267	28.159 ± 15.907	6.85 ± 2.404	25.619 ± 8.388	16.751 ± 11.871
PHO	0.036 ± 0.008	0.311 ± 0.086	0.884 ± 0.419	0.017 ± 0.007	0.386 ± 0.085	0.703 ± 0.478
KAR	0.059 ± 0.020	0.088 ± 0.020	0.082 ± 0.010	0.177 ± 0.018	0.059 ± 0.013	2.503 ± 4.230
Δ8D	0.400 ± 0.079	1.946 ± 0.203	1.555 ± 0.490	0.478 ± 0.031	1.478 ± 0.401	0.733 ± 0.590
ENR	0.147 ± 0.035	0.081 ± 0.008	0.567 ± 0.216	0.207 ± 0.073	0.095 ± 0.001	0.803 ± 0.677
PP	0.088 ± 0.003	0.116 ± 0.019	0.172 ± 0.057	0.049 ± 0.011	0.127 ± 0.042	0.312 ± 0.294
Δ9D	0.897 ± 0.093	0.671 ± 0.015	0.325 ± 0.303	0.889 ± 0.142	0.688 ± 0.115	0.720 ± 0.119
DGAT	0.091 ± 0.008	0.309 ± 0.048	0.147 ± 0.055	0.095 ± 0.023	0.344 ± 0.191	0.209 ± 0.036

Table S2. Total FA shown in ng/mg DW and %FA/mg DW in *Tetraselmis* sp. cultivated at different salinities (5 ppt, 10 ppt, 30 ppt, 40 ppt and 50 ppt), under nutrient stress (Day 2, nutrient replete; Day 4, nutrient deplete; Day 6, nutrient starved. Data represent mean values ± SDs from three independently-grown cultures.

Salinity	Day	Total FA ng/mL	%FA/mg DW
	2	147.57 ± 3.26	22.14 ± 0.49
5 ppt	4	63.09 ± 11.12	11.36 ± 2.00
	6	144.28 ± 22.72	17.31 ± 2.73
	2	130.45 ± 1.21	19.57 ± 0.18
10 ppt	4	83.53 ± 4.30	12.53 ± 0.64
	6	159.05 ± 9.37	19.09 ± 1.12
	2	96.66 ± 14.58	14.5 ± 2.19
30 ppt	4	122.44 ± 14.44	14.69 ± 1.73
	6	179.02 ± 36.55	16.11 ± 3.29
	2	97.16 ± 9.32	14.57 ± 1.40
40 ppt	4	144.39 ± 48.85	17.33 ± 5.86
	6	114.1 ± 98.91	15.4 ± 0.56
	2	113.72 ± 24.65	17.06 ± 3.70
50 ppt	4	88.55 ± 16.78	13.28 ± 2.52
	6	175.31 ± 47.33	21.04 ± 5.68

© 2014 by the authors; licensee MDPI, Basel, Switzerland. This article is an open access article distributed under the terms and conditions of the Creative Commons Attribution license (http://creativecommons.org/licenses/by/3.0/).
